# Supplementary material for: Seroprevalence and geospatial epidemiology of yaws: Evidence from Ghana
Source: PLoS Negl Trop Dis. 2025 Oct 16;19(10):e0013632. doi: 10.1371/journal.pntd.0013632 (PMC12543277; doi:10.1371/journal.pntd.0013632)
Supplement: S2 File — Maps were generated using ArcGIS 10.7.1 (Esri Inc., Redlands, California, USA). The shapefiles for Ghana and the various regions obtained from OpenStreetMap (https://www.openstreetmap.org/copyright, CC BY-SA 2.0) were utilized as data sources for plotting the maps. Map data from © OpenStreetMap. https://www.openstreetmap.org/copyright.’. (PDF) [file pntd.0013632.s002.pdf]

High-Low Clustering Report

Observed General G: 0.000257
z-score: 5.365348
p-value: 0.000000

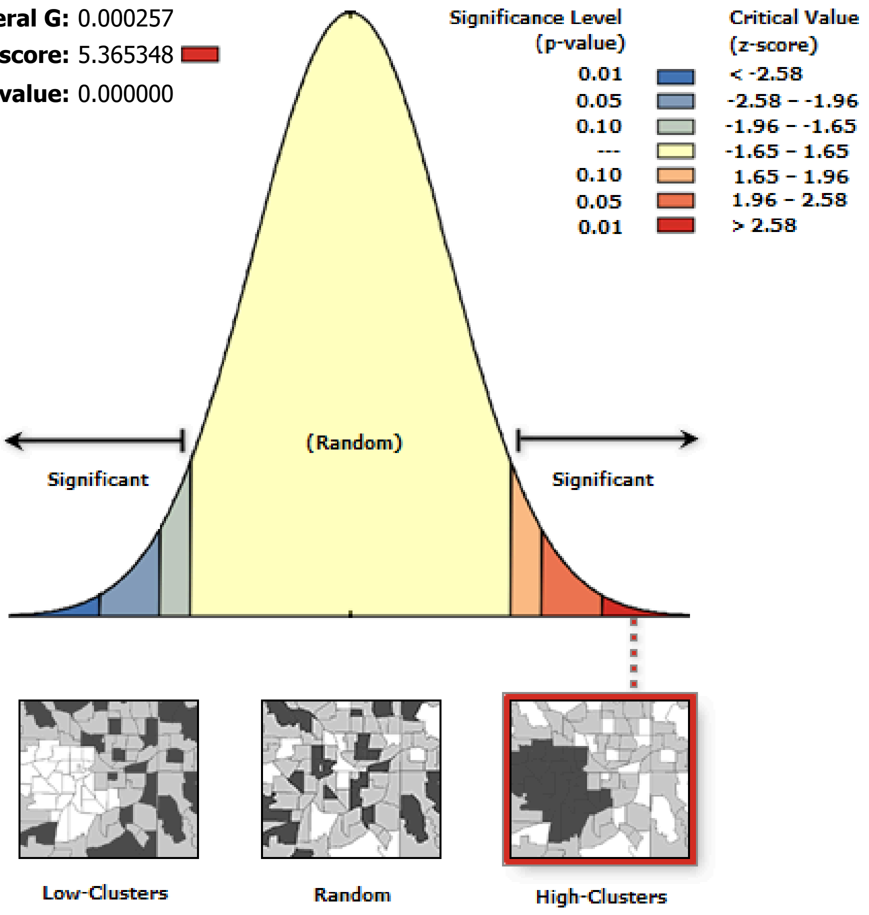

Given the z-score of 5.36534770156, there is a less than 1% likelihood that this high-clustered pattern could be the result of random chance.

General G Summary

**Observed General G:**	0.000257
**Expected General G:**	0.000036
**Variance:**	0.000000
**z-score:**	5.365348
**p-value:**	0.000000

Dataset Information

**Input Feature Class:**	newStudy_area_data
**Input Field:**	ADJUSTED_R
**Conceptualization:**	INVERSE_DISTANCE
**Distance Method:**	EUCLIDEAN
**Row Standardization:**	False
**Distance Threshold:**	10843.5891 Meters
**Weights Matrix File:**	None
**Selection Set:**	False
