# Supplementary material for: Seroprevalence and geospatial epidemiology of yaws: Evidence from Ghana
Source: PLoS Negl Trop Dis. 2025 Oct 16;19(10):e0013632. doi: 10.1371/journal.pntd.0013632 (PMC12543277; doi:10.1371/journal.pntd.0013632)
Supplement: S3 File — Maps were generated using ArcGIS 10.7.1 (Esri Inc., Redlands, California, USA). The shapefiles for Ghana and the various regions obtained from OpenStreetMap (https://www.openstreetmap.org/copyright, CC BY-SA 2.0) were utilized as data sources for plotting the maps. Map data from © OpenStreetMap. https://www.openstreetmap.org/copyright. (PDF) [file pntd.0013632.s003.pdf]

Average Nearest Neighbor Summary

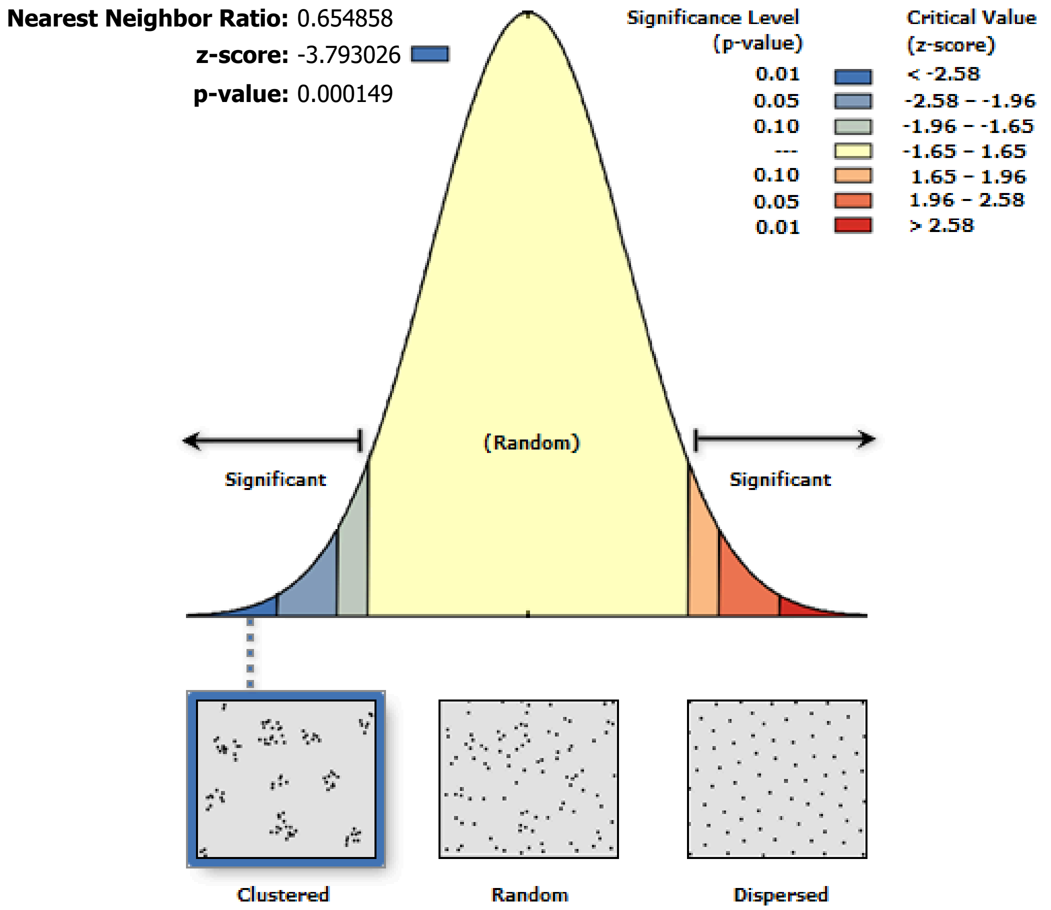

Given the z-score of -3.79302633067, there is a less than 1% likelihood that this clustered pattern could be the result of random chance.

Average Nearest Neighbor Summary

**Observed Mean Distance:**	3597.0775 Meters
**Expected Mean Distance:**	5492.9136 Meters
**Nearest Neighbor Ratio:**	0.654858
**z-score:**	-3.793026
**p-value:**	0.000149

Dataset Information

**Input Feature Class:**	newStudy_area_data
**Distance Method:**	EUCLIDEAN
**Study Area:**	3982717104.963760
**Selection Set:**	False
