# Supplementary material for: Seroprevalence and geospatial epidemiology of yaws: Evidence from Ghana
Source: PLoS Negl Trop Dis. 2025 Oct 16;19(10):e0013632. doi: 10.1371/journal.pntd.0013632 (PMC12543277; doi:10.1371/journal.pntd.0013632)
Supplement: Attachment — Submitted filename: Response to Reviewers.docx [file pntd.0013632.s005.docx]

23^rd^ September 2025

**Kumasi Centre for Collaborative Research**

**Kwame Nkrumah University of Science and Technology**

**Kumasi, Ghana**

**The Editor**

**PLoS NTD Journal**

Dear Sir,

**Re: ‘Seroprevalence and geospatial epidemiology of yaws: evidence from Ghana’ (PNTD-D-25-01271)**

My co-authors and I have taken note of the review comments and revised the manuscript as suggested.

We wish to submit the revised manuscript for publication in your esteemed journal. In the attached reply, the reviewer questions are in blue font and our responses are in red font.

We look forward to the next steps towards the publication of our manuscript.

Yours Sincerely,

Dr Yaw Ampem Amoako

Corresponding author

Point-by-point response to reviewer comments

**Journal Requirements:**

1) Some material included in your submission may be copyrighted. According to PLOSu2019s copyright policy, authors who use figures or other material (e.g., graphics, clipart, maps) from another author or copyright holder must demonstrate or obtain permission to publish this material under the Creative Commons Attribution 4.0 International (CC BY 4.0) License used by PLOS journals. Please closely review the details of PLOSu2019s copyright requirements here: PLOS Licenses and Copyright. If you need to request permissions from a copyright holder, you may use PLOS's Copyright Content Permission form.
Please respond directly to this email and provide any known details concerning your material's license terms and permissions required for reuse, even if you have not yet obtained copyright permissions or are unsure of your material's copyright compatibility. Once you have responded and addressed all other outstanding technical requirements, you may resubmit your manuscript within Editorial Manager. 
Potential Copyright Issues:

Comment: - Please confirm (a) that you are the photographer of Figure 3, or (b) provide written permission from the photographer to publish the photo(s) under our CC BY 4.0 license.

Response: Yes, I am the photographer of Figure 3.

Comment: - Figures 1, 4, 5, 6, and 7. Please (a) provide a direct link to the base layer of the map (i.e., the country or region border shape) and ensure this is also included in the figure legend; and (b) provide a link to the terms of use / license information for the base layer image or shapefile. We cannot publish proprietary or copyrighted maps (e.g. Google Maps, Mapquest) and the terms of use for your map base layer must be compatible with our CC BY 4.0 license.
Note: if you created the map in a software program like R or ArcGIS, please locate and indicate the source of the basemap shapefile onto which data has been plotted.
If your map was obtained from a copyrighted source please amend the figure so that the base map used is from an openly available source. Alternatively, please provide explicit written permission from the copyright holder granting you the right to publish the material under our CC BY 4.0 license.
If you are unsure whether you can use a map or not, please do reach out and we will be able to help you. The following websites are good examples of where you can source open access or public domain maps:
* U.S. Geological Survey (USGS) - All maps are in the public domain. (http://www.usgs.gov)
* PlaniGlobe - All maps are published under a Creative Commons license so please cite u201cPlaniGlobe, http://www.planiglobe.com, CC BY 2.0u201d in the image credit after the caption. (http://www.planiglobe.com/?lang=enl)
* Natural Earth - All maps are public domain. (http://www.naturalearthdata.com/about/terms-of-use/).

 Response: Maps and spatial analysis were generated by the authors using ArcGIS 10.7.1 (Esri Inc., Redlands, California, USA). Publicly available shapefiles from OpenStreetMap (*www.openstreetmap.org*) were used for base mapping. Information on the terms of use / license information for the base layer images or shapefiles were provided in all the figure legends.

**Reviewers' comments:**
**Methods:**

Reviewer #1: The methods are mostly well described and appropriate, but how prevalence and seroprevalence is addressed needs attention. Comments for consideration:
1. Clarification on the different tests being used throughout the text. It is difficult to follow in certain sections whether DPP or RDTs are being referred to.

Response: In the section on statistical analysis, we included definitions for yaws prevalence in each district (line 243-244), RDT seroprevalence and DPP seroprevalence (line 245-251). In addition to these definitions, we have included references to the specific tests (DPP or RDT) within the manuscript in areas to address the review comment. Furthermore, Table 2 has details of the information.

2. In the statistical analysis section, seroprevelance should be considered to have the whole study population as the denominator, not just clinically-suspected cases that were screened. Or at least a comment that the seroprevalence is biased / limited due to only reporting seroprevalence in clinically-suspected cases.

Response: Thank you for your comment. We would like to clarify that, in Table 2, all prevalences were calculated using the whole study population or the relevant subgroup populations as denominators. In contrast, seroprevalence in this study was defined as the number of participants testing positive with the serological test expressed as a percentage of the total number tested with the serological test. Since only those clinically suspected were tested, this naturally limits the denominator to that group. However, if seroprevalence were recalculated using the whole study population as the denominator, the rates would be the same as that of the prevalence (for the DPP seroprevalence). We have noted this definition clearly to avoid any ambiguity. Furthermore, we have added the following sentences to the limitations section:

‘Due to limited availability of serological test kits, only clinically suspected participants and individuals reported to have had untreated but resolved yaws-like lesions were tested. The estimates may not fully represent the true seroprevalence in the general population and may be biased toward higher seropositivity missing other possible latent infections. Nevertheless, focusing on clinically suspected individuals provides important insights into the serological profile of those most likely to be actively transmitting the disease, and is valuable for guiding targeted surveillance and intervention in endemic settings where universal testing is not feasible.’
3. The impact of serological cross-reactivity should be mentioned.

Response: We have done as suggested by adding the following sentences to the limitations section (line 539-545):

‘Cross reactivity between syphilis and yaws make it difficult to differentiate between the two diseases based on serology alone. This lack of specificity, along with the potential for both diseases to coexist in the same areas, complicates epidemiological surveillance and diagnosis and a reliance on serologic tests alone could result in an overestimation of prevalence. In this study, we combined clinical examination findings and serology to improve diagnostic accuracy. While molecular tests such as polymerase chain reaction (PCR) offer higher specificity, they were deemed impractical in this field survey.’

4. Consider adjusting the prevalence estimated for clustering at the school level.

Response: We have done as suggested. We have added to the data analysis section the following sentences:

‘Adjusted prevalence ratios were calculated using RStudio version 2023.12.0.369 (RStudio: Integrated Development Environment for R. Posit Software, PBC, Boston, MA, USA) with the survey package to account for differences in school population sizes, providing a more reliable measure for spatial comparisons.’

The results are now reflected in figures 4, 5, 6 and 7.

Reviewer #2: The manuscript does not provide information on how the sample size was determined. Please include details on the sample size calculation or justification, and specify the method or assumptions used.

Response: This was a school survey. We stated in the methods the reasons/ assumptions behind the selection of the choice of districts (line 148-151), schools and age range of individuals (line 157-164). All persons who met the inclusion criteria and had consent provided were recruited. The section reads (line 157-164):

‘This was a cross-sectional study conducted between May and December 2024. In each district, the choice of schools to visit was guided by the District Health Management Team (DHMT), relying on historical reporting trends for yaws within the area. In Wassa Amenfi East, a total of 33 basic schools in 29 communities were visited, while 4 basic schools in 4 communities were visited in Aowin district (Figure 1). Based on the known epidemiology of yaws, children in the basic schools who typically fall in the key transmission age [4] were included in the study, along with children aged 1-5 years who, although typically not enrolled in basic schools, may be found in kindergarten schools and are key for ongoing disease transmission [18].’

**Results:**

Reviewer #1: The analysis does represent the analysis plan and for the most part well written, but how prevalence and seroprevalence is addressed needs attention. Comments for consideration:
5. Throughout the results section it would be helpful to report n/N (%) more consistently as it can be difficult to follow what the numerator or denominator is.

Response: These were included in the relevant tables but have been restated in the appropriate areas of the text as well.
6. There is repetition in the first two paragraphs of the results regarding prevalence. This can be streamlined.

Response: Done, the section now reads:

‘Of the children examined clinically, 13% (1502/11505) either showed lesions suggestive of yaws or had previously had similar lesions that resolved without treatment.’
7. The reporting of prevalence / seroprevalence is hard to follow. There are many different ways seroprevalence is reported (for RDTs and DPP) within different sub-cohorts (e.g., DPP seroprevalence in RDT positive cases).

Response: This has been clarified in the definitions of the and methods section.
8. Table 1 - please be consistent with decimal places.

Response: Done as suggested.
9. Table 1 - what does the asterisk refer to?

Response: The asterisk has been deleted
10. Table 3 - please include totals for primary, secondary and latent stage.

Response: This has been done as suggested.

Reviewer #2: The presented analysis aligns with the stated objectives, and the results are clearly detailed. Figures and tables are of acceptable quality and effectively support the findings.

Response: Thank you.

**Conclusions:**

Reviewer #1: The conclusions are supported by the data but there are issues with how prevalence / seroprevalence is being reported. Additional comments for consideration:
11. Line 523 mentions environmental and socioeconomic drivers of transmission, but they were not evaluated.

Response: Thank you. We have revised and provided a reference for the statement.

Reviewer #2: There are no significant issues here. The data support the conclusions.

Response: Thank you

**Editorial and Data Presentation Modifications?**

Reviewer #2: Only minor editorial adjustments are required, primarily to enhance the clarity of tables and to ensure that denominators are clearly indicated in the results. The figures and tables are otherwise of acceptable quality. I therefore recommend a minor revision.

Response: Many thanks for your review.

**Summary and General Comments:**

Reviewer #1: This is an important piece of research but further thought on how to approach prevalence / seroprevalence is needed, that is inclusive of the whole study population.

Response: Thank you. Answers to specific review comments have been provided above.

Reviewer #2: This study makes a valuable contribution by combining seroprevalence data with geospatial analysis of yaws in Ghana, tackling an important but under-researched neglected tropical disease. The methods and analyses are appropriate, the results are clearly presented, and the public health significance is well explained. The only aspect needing clarification is the sample size determination. Overall, I recommend the manuscript for publication with minor revisions.

Response: Thank you. Answers to specific review comments have been provided above.
