# Supplementary material for: Seroprevalence and geospatial epidemiology of yaws: Evidence from Ghana
Source: PLoS Negl Trop Dis. 2025 Oct 16;19(10):e0013632. doi: 10.1371/journal.pntd.0013632 (PMC12543277; doi:10.1371/journal.pntd.0013632)
Supplement: Attachment — Submitted filename: Response_to_Reviewers_auresp_2.docx [file pntd.0013632.s006.docx]

10^th^ October 2025

**Kumasi Centre for Collaborative Research**

**Kwame Nkrumah University of Science and Technology**

**Kumasi, Ghana**

**The Editor**

**PLoS NTD Journal**

Dear Sir,

**Re: ‘Seroprevalence and geospatial epidemiology of yaws: evidence from Ghana (PNTD-D-25-01271R1)’**

My co-authors and I have taken note of the review comments and revised the manuscript as suggested.

We wish to submit the revised manuscript for publication in your esteemed journal. In the attached reply, the reviewer questions are in blue font and our responses are in red font.

We look forward to the next steps towards the publication of our manuscript.

Yours Sincerely,

Dr Yaw Ampem Amoako

Corresponding author

Point-by-point response to reviewer comments

**Reviewers' comments:**
Reviewer #2: My concern in the last edition was the non-inclusion of the sample size calculation; unfortunately, this has still not been addressed in the current revision.

Response: Many thanks for your comment.

In response to the review comment, we have added the following sentences to the methods section (line 164-166):

‘Due to the focal endemicity and uneven distribution of yaws as a disease, no formal sample size calculation was done, and the sample size was based on the convenience of sampling in the selected schools.’
